# Integrated Ground-Based SAR Interferometry, Terrestrial Laser Scanner, and Corner Reflector Deformation Experiments

**DOI:** 10.3390/s18124401

**Published:** 2018-12-12

**Authors:** Xiangtian Zheng, Xiaolin Yang, Haitao Ma, Guiwen Ren, Keli Zhang, Feng Yang, Ce Li

**Affiliations:** 1 School of Mechanical Electronic & Information Engineering, China University of Mining and Technology (Beijing), No. Ding-11, College Road, Haidian District, Beijing 100083, China; aforest0459@foxmail.com (X.Z.); zhang_keli@163.com (K.Z.); yangf@cumtb.edu.cn (F.Y.); celi@cumtb.edu.cn (C.L.); 2 China Academy of Safety Science and Technology, No. 32, Beiyuan Road, Chaoyang District, Beijing 100012, China; maht@chinasafety.ac.cn (H.M.); rgwen104@163.com (G.R.)

**Keywords:** ground-based synthetic radar (GB-SAR), terrestrial laser scanner (TLS), corner reflector (CR), deformation experiment, geometric mapping

## Abstract

An integrated sensor system comprised of a terrestrial laser scanner (TLS), corner reflectors (CRs), and high precision linear rail is utilized to validate ground-based synthetic aperture radar (GB-SAR) interferometric micro-displacement measurements. A rail with positioning accuracy of 0.1 mm is deployed to ensure accurate and controllable deformation. The rail is equipped with a CR on a sliding platform for mobility. Three smaller CRs are installed nearby, each with a reflective sticker attached to the CR’s vertex; the CRs present as high-amplitude points both in the GB-SAR images and the TLS point cloud to allow for accurate data matching. We analyze the GB-SAR zero-baseline repeated rail differential interferometry signal model to obtain 2D interferograms of the test site in time series, and then use TLS to obtain a 3D surface model. The model is matched with interferograms to produce more intuitive 3D products. The CR displacements can also be extracted via surface reconstruction algorithm. Finally, we compared the rail sensor measurement and TLS results to optimize coherent scatterer selection and filter the data. The proposed method yields accurate target displacement results via quantitative analysis of GB-SAR interferometry.

## 1. Introduction

Deformation monitoring and displacement measurement of unstable slopes and buildings are crucial for surface observation and disaster prevention. A variety of measuring sensors have been used to determine explicit shape variations and regional targets deformation patterns in time and space. Current sensors can be divided in contact or non-contact mode categories. The former includes the inclinometer, strain gauge, optical fiber sensor, terrestrial laser scanner (TLS), total station (TS), global positioning system (GPS), and other monitoring systems placed on the surface or embedded in the target body [1]. Contact measurement technologies generally troubled by small coverage and risky installation in hazardous areas. Non-contact sensors mainly include prismatic-free total station, real-aperture radar (RAR) [2,3], and interferometric radar (IN-SAR) [4]. 

Unlike other remote sensors, IN-SAR provides continuous spatial coverage and is relatively insensitive to the surrounding environment, facilitating long-term overall analysis and prediction. IN-SAR may be space-borne, air-borne, or ground-based. Space-borne and air-borne IN-SAR sensors merit good spatial resolution, short sampling time intervals, and high accuracy deformation monitoring. Ground-based synthetic aperture radar (GB-SAR) interferometric deformation measurement is a newer technology, exploits the same principle used in spaceborne. GB-SAR installations are more flexible in geometry arrangement when monitoring small-scale deformation phenomena, which has been successfully applied in early warning for landslides [5], subsidence observation [6], glacier displacement observations [7], building structure stability monitoring [8], bridge safety evaluation [9,10], earthquake deformation field realization [11], snow covered avalanche identification [12]. 

This paper focuses more on the GB-SAR experimental methodology of slope deformation monitoring. Although many advantages of GB-SAR monitoring have been found, and the application has shown GB-SAR monitoring technology has been well developed, however, some data of GB-SAR is still found not stable as contact-mode monitoring instruments during the long-term application or used for short-term emergency management. The principle utilized is similar between GB-SAR and space-borne InSAR, namely extracting the small-scale deformation of the monitoring area through interferometry in the phase, in which the interfered phase quality plays the essential role. Factors affecting the phase’s quality of InSAR involve: ① Baseline decorrelation; ② Decorrelation caused by inconsistent spectrum centroid; ③ Temporal decorrelation, including the decorrelation caused by temperature and atmospheric conditions during the re-visit period [13]. Errors may accumulate over time, so it is necessary to validate the deformation measurement before long-term monitoring to ensure stability and reliability [14]. Tarchi et al., for example, carried out a campaign on monitoring the landslide in Tessina. A vector network analyzer (VNA) based GB-SAR was compared with motorized theodolite and electronic distance meter (EDM) which were conventional topographic instruments. Time-series displacement of two benchmarks whose positions were pre-measured within the illuminated area was gathered. The utilized focusing processor could be performed on an arbitrary set of points, thus, two benchmarks could be easily located in GB-SAR displacement map. The variance of the two resulting fitted curves was investigated benchmarks [15]. However, the deformation of complex landslide motion was not controllable, which will generate wrapped displacements. Optical results were used to unwrap the phase of GB-SAR at the benchmarks, but deformation at other locations were not further verified. Pieraccini et al. projected GB-SAR interferograms with other data onto a Digital Elevation Model (DEM) obtained by In-SAR. They found that DEM is not consistent with GB-SAR; the projection result was only applicable for qualitative verification [16]. They later improved this scheme for the co-registration of topography and interferogram data with TLS deformation measurements in an urban area. However, TLS precision, with a ranging error of 8 mm @ 100 m, produced a centimeter-level disparity among result sets [17]. Lombardi et al. projected time-series displacement maps onto higher accuracy point cloud (resolution of 0.008° in the 100 m range) when monitoring for a landslide where workers were restoring the corrupted pipelines [18]. Although it was not detailed in how to obtain higher interferometric phase quality, the most critical areas were localized with respect to the work activities and the GB-SAR detected displacements were 3D visualized via merging with TLS 3D model. Thus, they succeeded in developing a simplified early warning system via control points selection and tracking the deformed points with the rainfall events. 

More controlled validation schemes have been designed to verify deformation in time series. Lingua et al. used a metal disk with a worm gear motor engine to produce controllable displacement (0.01 mm) in average 120 m distance from a monitoring station in Florence, Italy. They attempted to estimate GB-SAR measurements but the DEM matching accuracy proved problematic as the metal disk was ambiguous on the terrain point cloud [19]. Qu et al. used a 7 GHz (intermediate frequency) GB-SAR system to observe deformation in a metal ball and bar, but the displacements were manually caused and not precisely measured [20]. Yang et al. conducted a campaign between GB-SAR and total station in the same location and made the two groups shared same data interval to test several radar targets with presence of prisms. The results were co-registered under the total station coordinate system; however, the movements were not significant (displacements were less than 1 mm) [21]. And authors [22] proposed a method to verify the deformation of a controllable field methodology, GB-SAR measurements were validated by an automatic robot which was also a topographic technique during a bollard pull trial on deformation response of a pier, 12 co-located CRs and surveying prisms (SP) were utilized. The pull trial made all the targets differently deformed. Thus the deformation patterns could be easily corresponded with pulling force measured by a load cell. 

The aim of this paper is to describe a standard procedure used for testing the deformation monitoring capability of GB-SAR system produced by us is described in this paper, which is also adopted in testing the applicability of radar for its main application in monitoring of slope and landslide in emergency conditions. Authors co-designed and conducted an outdoor controlled field test to validate GB-SAR displacement measurement in a relatively small area. We used a Ku-band GB-SAR called S-SAR developed by our research lab in China Academy of Safety Science and Technology (CASST) (http://www.chinasafety.ac.cn/) for the quantitively study. The main application objects of S-SAR are mine slope and landslide disaster emergency monitoring. Ku waveband radar system covers several characteristics, the easily achievable hardware performance, high spatial resolution images as well as high sensitivity to small changes in ground features. In addition, atmospheric influence (such as temperature, pressure and moisture) can weaken the high-frequency signal, which can reduce monitoring accuracy and bring difficulties to the deformation analysis, and further affect the apprehension and prediction on slope disasters. However, high sensitivity of Ku waveband also causes added burdens, therefore, how to eliminate the small deformation caused by environmental changes plays a vital role in the data processing [23]. Existing deformation validation schemes merit improvement in a few specific aspects. Although some controllable steps were set up, there are still many interference factors. Therefore, controlled field scheme could be redesigned. The displacement map could be matched accurately with a high-precision (mm level) DEM or DSM model for intuitive and credible results validation as [18], differently we use the 3D mapping to analysis abnormal deformed areas in the experiment. And, the geometric parameters of displacement generation device accurately calculated and precisely controlled. Comparison sensors accuracy should be also at millimeter level to generate validation data (i.e., the same measurement range). The main purposes are explicit the relationship between GB-SAR displacement map and targets, locate the pixels that deform intensively, eliminate abnormal areas and reduce the factors caused inaccuracies. Besides, although there are many differences between the experiment with close range and small scene and that with long distance and large scene, a variety of problems can also be solved in the controllable deformation experiment with close range. What’s more, GB-SAR deformation monitoring coverage can be required to the range from 10 m to 5000 m. Therefore, the scene with close range is firstly selected to conduct the experiment in this paper.

The rest of this paper is arranged as follows: Section 2 briefly presents our experimental scheme and test site. Section 3 explains the GB-SAR interferometric deformation measuring model in the controlled field. Section 4 discusses the relationship between the GB-SAR sensor and TLS point cloud, and proposes geometric mapping method using the feature points. We also present a TLS displacement extraction method based on surface reconstruction and geometrical relationship analysis. A comparative analysis and optimization of results are also provided in Section 4. In Section 5 discusses to validate the merit of the proposed scheme, and Section 6 concludes the paper.

## 2. Experimental Scheme and Test Site

**Figure 1:**
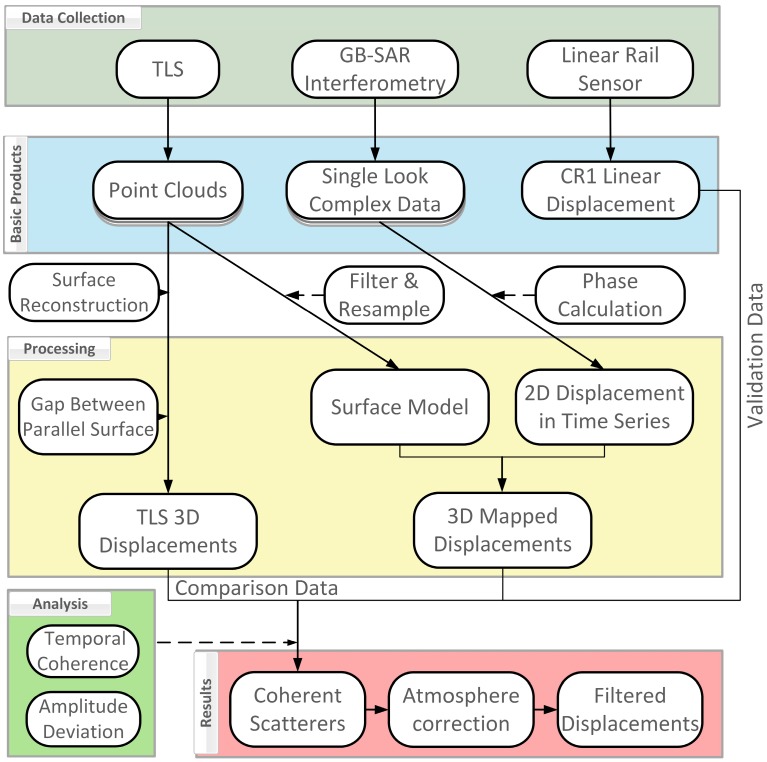
Experimental scheme: integrated sensors for controlled field experiment operative work flow (modified according to [24]).

Outdoor experimental scheme, as shown in Figure 1. Artificial targets were placed 10 m away from the GB-SAR, mainly including four CRs, a linear rail (about 30 m from GB-SAR), and a tank. Reflective stickers were pasted on the side-walls and each CR vertex (except for CR4). Two more stickers were placed on the middle of each transmitting and receiving antenna aperture, and then the antennas were motored back to the initial rail location. We used the TLS to scan the scenario once, then moved the antennas to the rail terminal and ran the TLS again. We next co-registered the TLS point clouds using control points to establish the surface model (DSM) and a sticker recognition algorithm to identify the target and control point coordinates. We ran the GB-SAR repeatedly in 10 min intervals to obtain 2D complex imaging figures, coherent figures, and interferograms all in time series. When the GB-SAR was paused, the CR1 rail generated linear displacement under software control and recorded the displacement measured by the rail sensor. The TLS also scanned the scene at the same interval. Interferograms were projected onto the DSM and evolved into 3D deformation maps as we calculated the displacement in the time series of each feature point by surface fitting. Finally, we integrated the TLS displacements and rail sensor results to accomplish coherent scatterers and final filter processing.

**Figure 2:**
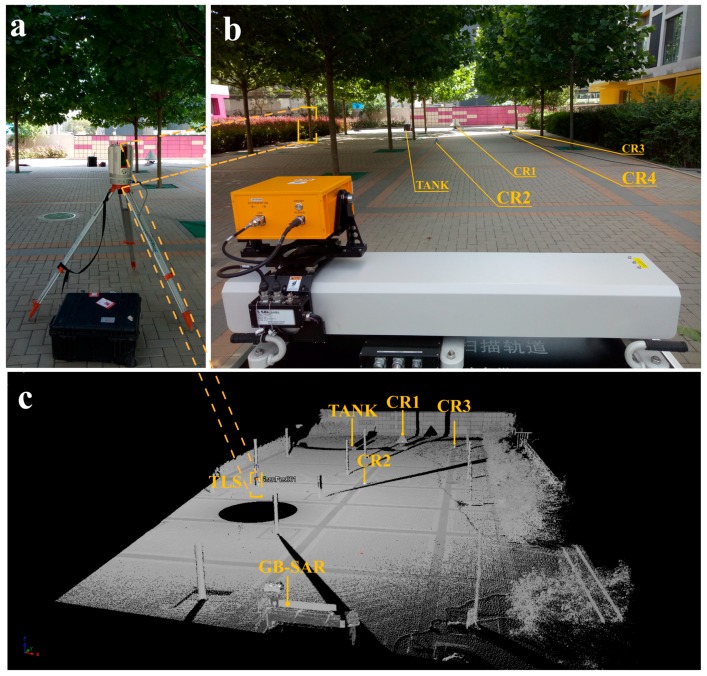
(**a**) Tripod-supported TLS placement; (**b**) Test site from the perspective of GB-SAR under bore-sight model; (**c**) Processed point cloud where various gray values represent various laser beam reflection intensities.

The test site is shown in Figure 2. The scenario was checkerboard-like to conveniently estimate the relative positions of targets. The GB-SAR system was placed on a wheel-less box to fix the antennas at a certain height. The bottom of the box is flat to ensure a stable platform. The scene was mapped using a VZ-2000i TLS (RIEGL Co., Horn, Austria) [25]. About 7.5 million points were collected in each scan after scene segmentation. The TLS station position is shown in Figure 2b,c. Figure 2c shows the relative position between the GB-SAR and each target. The circular vacancy is the “blind area” of the TLS station location scanning, the central position of which is the TLS axis. The main artificial targets are small corner reflectors (CR2, CR3, CR4), a large corner reflector (CR1) displacement test rail, and a black tank (TANK). Reflective stickers serve as strong amplitude points in TLS measurement. CR4 was obscured by a tree trunk in the TLS observation, so it was not identified in the point cloud. CR4 was arranged for geometric mapping validation. Generally, if the 2D image pixels representing CR4 are mapped to 3D space exactly in the shadow zone, the projection results are credible. There were relatively stable natural targets in the site including masonry ground, trunks and a brick wall. To this effect, the test site indirectly simulates the observation of deformation under natural conditions. There is dense vegetation on both sides and the upper part of the image. As shown in Figure 2b, the facade of the buildings on the left and right sides was not covered by the radar azimuth beam. The point cloud of the trunk (Figure 2c) was retained to verify the influence of the trunk swing, although the oscillation frequency is large. Relatively few pedestrians or vehicles passed through the scene during the experiment, so these effects were ignored.

**Figure 3:**
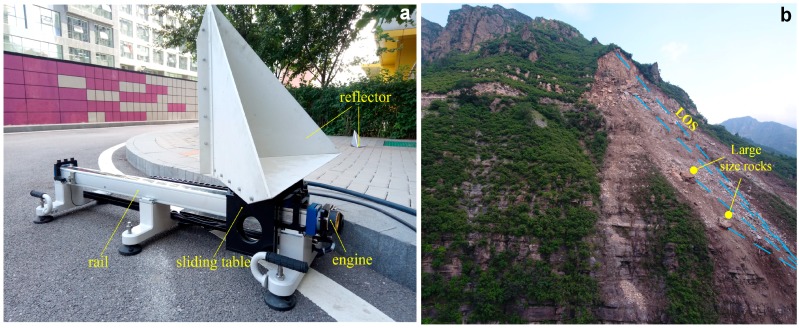
(**a**) Linear sensor system employed to generate controlled displacement; (**b**) A mountain rockslide, the movement of corner reflector simulates the displacement of giant rocks on the surface of the rockslide accumulation zone. Blue dashed line shows the LOS direction, and yellow line marks two large size rocks on the slope surface.

We optimized the layout and physical properties of CRs for the proposed scheme. CRs were used for deformation verification though they may cause high regional echo coherence. CRs are strong scatterers which present as “bright spots” in radar images; the size of their amplitude peak pixels is approximately a resolution unit. The CRs we used were made of aluminum alloy plates with inherently high electrical conductivity.CR1 was totally coated with conductive cladding material in order to provide high signal to noise ratio (SNR) results. According to the backscatter features, the largest CR’s reflective cross section area (RCS) (denoted by *σ_max_* in dbsm units) ensures that the normal of the CR’s mouth surface is approximately parallel with the incident direction of radar waves. The maximum RCS is as follows [26]:

(1) $$\sigma_{max}=\frac{4\pi l^{4}}{3\lambda^{3}}$$

where *l* represents the inside edge length of the CR and *λ* is the length of the radar waves. We increased the bottom vertex of the CRs so that it would reflect more strongly than surrounding objects (natural ones). Each CR is equipped with a reflective sticker 5 cm in circle diameter with a 3-mm diameter center hole. We made sure that the circle was pasted on parallel to the mouth surface of the angular reflector and tangent to the three respective faces. When the line of sight (LOS) is parallel to the normal direction of the oral surface, the corner points of the angular reflector are fully visible. The incident angles of CR1 and CR2 had the maximum reflective cross-sectional area while CR3 and CR4 did not. The composition of the deformation verification system is shown in Figure 3.

Different from the monitoring method of benchmark displacement in the [15], corner reflectors are used to simulate the giant rocks near the slope, which forms a certain angle with the slope and have a strong reflection on the radar waves. The complicated slope surface displacement is non-linear. Collapse accumulation includes the upper accumulation part and the lower residual dangerous rock mass, and under the condition of little lithological characters, the difference in scattering characteristic is mainly from the monitored geometric and morphological difference. The strong reflection points of radar waves are from the irregular surface of giant rocks or residual rock mass of the accumulation, while there is a close relation between the reflection wave intensity and incident direction. However, the slide of giant rocks along the slope surface can be recognized as linear during the two-view image scanning interval of GB-SAR and the deformation monitored by GB-SAR is the component of the actual deformation along the LOS direction. As showed in Figure 3b from a road slope collapse emergency monitoring activity, a certain angle was formed between the giant rock and the slope surface, and the volume of the giant rock was 3 × 3 × 5. And the corresponding pixels on the radar reflection intensity image were also represented as amplitude strength. For the landslip and the dangerous side slope, regional collapse may not cause the overall slump; however, the accurate location and scope definition of dangerous regional collapse rock mass and following the relation of its displacement with time serve important roles.

We oriented the rail parallel to the ground or placed it at a certain angle by adjusting the screws in the system, where a single ring length was 2 mm. The three T-shaped bases are made of metal materials which impact the interferometric phase of GB-SAR, so they were buried underground. The system was placed behind a high step and the base screws turned to the bottom and leveled so as to achieve the same effect as embedding them underground. The rail was driven by a motor and belt with ball screw mechanical transmission compositions inside. Precise movements were realized via software and driver. A grating ruler and position sensor were installed in the sliding table. CR1 was rigidly connected to the table by screws. These compositions were altogether utilized to achieve sub-millimeter displacements. The linear system parameters are shown in Table 1.

## 3. GB-SAR Working Mode and Interferometry

### 3.1. GB-SAR Zero-Baseline Interferometric Working Mode

The GB-SAR system transmits and receives step-frequency continuous waves (SFCW) in the range direction and forms a synthetic aperture in the azimuth direction via beam-forming algorithm. A back projection (BP) algorithm is applied in the imaging process. A dual-antenna interferometor was installed on a linear rail: one antenna transmitted electromagnetic waves and the other received reflected waves. The antennas were driven by a motor on the rail, moving in a “go-stop-go” mode. The interferometer moved from the rail initial position to a set position to form a synthetic aperture with length denoted by $L_{s}$; *L* is the effective length of the rail, so $L_{s}\leq L$. The antennas moved at a fixed time interval. Every stop represents one azimuth sampling point in a series of N points. The SFCW frequency hopping number is *K*. Original echo data are represented by a K×N complex array. According to the principle of GB-SAR interferometric measurement, the phase variation of each pixels in master image and slave image are compared in the continuous scanning mode, while the phases of each pixel of the same image are not compared during scanning process. Therefore, it is assumed that the interference phase of each pixel of the same scene image has no change. So, during one scan, we assume that no relative displacement occurred between targets and the GB-SAR. The main characteristics of the GB-SAR are shown in Table 2. 

**Figure 4:**
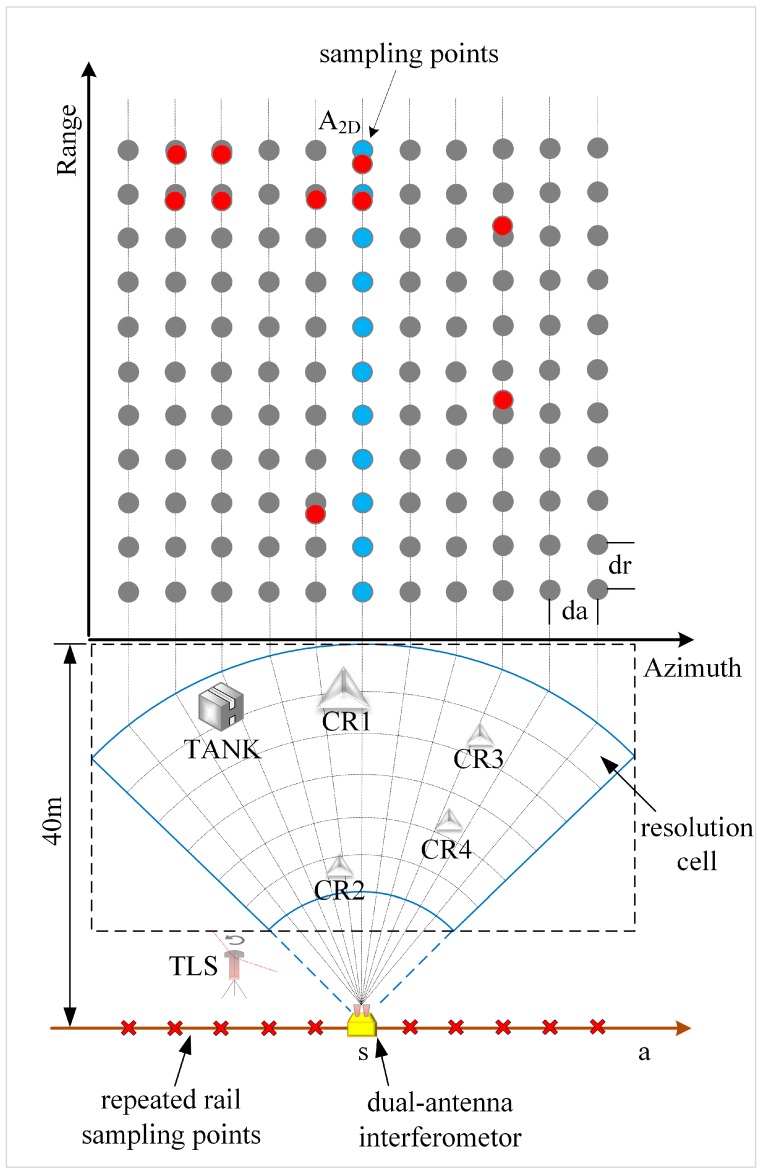
Signal spatial distribution of main targets and GB-SAR image spatial mapping relation.

In fact, the combination of these monitoring parameters is pretty important for the analysis on the object’s deformation. But, in this experiment, only common parameters in the side slope monitoring process are chosen to ensure a relatively high spatial object resolution (such as giant rock or open-pit slope steps), and a high echo quality so that accurate deformation can be obtained after the post-treatment, which, however, is not the main point of this paper. Figure 4 shows the spatial relationship between each artificial target and the GB-SAR as well as the relationship between GB-SAR images in polar coordinates and right-angle coordinates. The sector shape is the beam coverage area. Each grid represents a resolution cell and the radial grid length is equal to the range resolution $\delta_{r}$.

(2) $$\delta_{r}=\frac{c}{2B}=\frac{c}{2(K-1)\Delta f}$$

where *c* represents the propagation speed of electromagnetic waves, *B* denotes the bandwidth, and *K* is illustrated in Table 2, which is the number of transmitted frequencies; Δ*f* is the frequency hopping interval and the radial length is a fixed value. Δ*f* is to obtain an unambiguous range *R_U_* twice the distance of the farthest target the radar can detect. For the characteristics in Table 2, *R_U_* is 3 km. To reduce the effect of side lobes in range and azimuth synthesis, data are corrected by means of a window function (Kaiser function with coefficients 3 and 6) [23].

**Figure 5:**
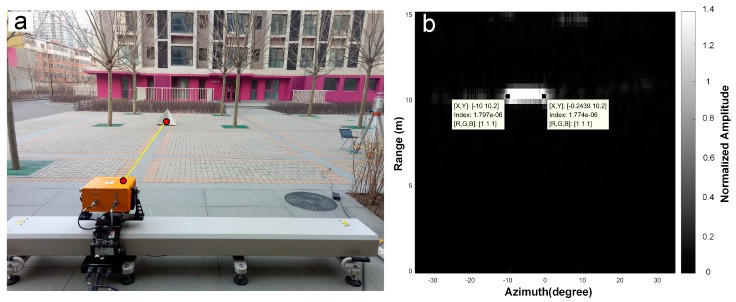
Another test for “artefact” using a 2 m GB-SAR. (**a**) Shows the set up of the test, CR1 was about 10m from GB-SAR and (**b**) is the amplitude image of the CR which is shown as long bright spot.

The total accumulative displacement of CR1, designed in experiment plan is smaller than *δ_r_* which is the range resolution, in case of the problem that CR1 becomes two targets for GB-SAR. GB-SAR is not using the full azimuth aperture, echo signals of a single point target within antenna beam appear in each of the linear rail and in raw data of all frequencies. At this moment, the image is in a defocusing state. For each image element of the original data, the ideal point object has the same strength and the phase is the function of the emitting frequency and the sensor in the orbital position. The observed values of strength and phase need to be converted to the grid with a spatial resolution, and the image focusing involves distance and azimuth direction focus, which embodies the image with distance- and azimuth direction spatial resolution. In the azimuth direction, the array element spontaneously self-collects and the beam width is half of the actual beam [27], which is the radian value clamped at the point that the power of the beam’s main lobe drops by 3 dB. The beam width can be retrieved via:

(3) $$\theta_{BW}=\frac{\lambda_{c}}{2L_{s}}$$

where *λ_c_* denotes the middle frequency wavelength and $L_{s}$ is the synthetic aperture length. As shown in Figure 4, the CR1 and TANK are larger in size than one resolution cell, while CR2, CR3, and CR4 are smaller than one resolution cell. dr and da are the length and width of a GB-SAR image pixel, respectively. dr is consistent with the range resolution $\delta_{r}$ and da is equal to the space length of the beam width of the projection azimuth from the center of the scene. Thus:

(4) $$da=\theta_{BW}R_{ref}$$

where *R_ref_* is the distance between the scene center point and the phase center (red point in Figure 5a), and da is an approximation. In the experiment, da was about 22 cm and dr was 30 cm. I(a,r) represents the pixel complex value, where r is the range and *a* is the azimuth. I(a,r) can be denoted as:

(5) $$I(a,r)=\sum_{n}e^{\left(-j4\pi \cdot r_{n}/\lambda_{c}\right)}\cdot psf(a-a_{n},r-r_{n})$$

where $r_{n}$ represents the range of the nth target, $a_{n}$ denotes the nth target azimuth length to (0,0), and $\lambda_{c}$ is the intermediate frequency wavelength, psf(·), on behalf of the point spread function (PSF), is the impulse response of the imaging system for a low-pass filter point target. The results of Equation (5) not only contain amplitude and phase information, but also represent the physical properties and geometric parameters of the imaging scene. For Figure 4, the grey dot refers to the grid pixels, and the blue dots are the middle axis of the image while the red dots are the ideal pixel positions in the image space of each target in the experiment. Actually, the pixel grid coordinates of the image are usually not consistent with the position of the real surface object; therefore, there is a deviation between the red dot and the grey dot. In the image processing, the azimuth width of the actual observation scene is often longer than the azimuth orbital length L, but the difference is usually small, and zero fill along the azimuth direction is needed for both two conditions [15]. The principle is to ensure an equivalent length of signal along the azimuth between the experiment and the actual situation, otherwise, the azimuth image blurring will occur and affect the SAR image quality. Due to the experiment conducted in the regional observation area with close distance, the attenuation of the electromagnetic wave along distance caused by the changes of observation distance cannot be ignored, and even the image will be defocused seriously. In addition, for natural strong scatterer or artificial object like corner reflector, the energy may diffuse in the image space, and the phenomenon of relatively longer extension length in the azimuth and the relatively diffused that in the distance direction, in which the artifact will be produced, the experiment to test artefact as shown in Figure 5.

Before the field test, another experiment was conducted which utilized the same focusing processing parameters but different in Table 2. As shown in Figure 5, another length of linear rail was utilized with a synthetic aperture of 2 m. CR1 was placed about 10 m from GB-SAR. According to Equation (4), the azimuth resolution is better than the 1 m linear rail. To meet the demand of deformation generation near real time, a short time interval of GB-SAR should be ensured, and the approximation algorithm is used here for imaging. To ensure it available for both close and distant scenes, phase protection and focusing should be conducted fast. Actually, the ground radar should meet the demands of imaging under wide angle in both close and distant scenes. Strictly speaking, the preset inner imaging algorithm of the system is designed for rapid imaging, and an imaging method based on the pseudo-polar coordinate system [28] has been used, which is involved in this paper for the focus on deformation. Artefacts, in fact, the problem in the case of long range monitoring also is not very good eliminated, we set up another remote experiment in a open-pit mine, also found on average range of 800 m. CR was shown as strong scatterer, its size should have affected only one pixel, the actual impact on the number of pixels was a lot more to one unit, according to the Equation (4) to calculate reach to over 10 m in the azimuth direction.

### 3.2. Experimental Displacement Extraction

**Figure 6:**
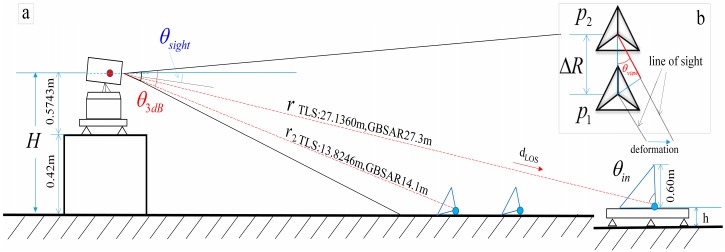
(**a**) Interferometric LOS displacement extraction in range profile, where *r* denotes the distance from the phase center to CR1 and *r*_2_ represents the range between the phase center and CR2; (**b**) CR1 deformation illustration, moving from *p*_1_ to *p*_2_.

**Figure 7:**
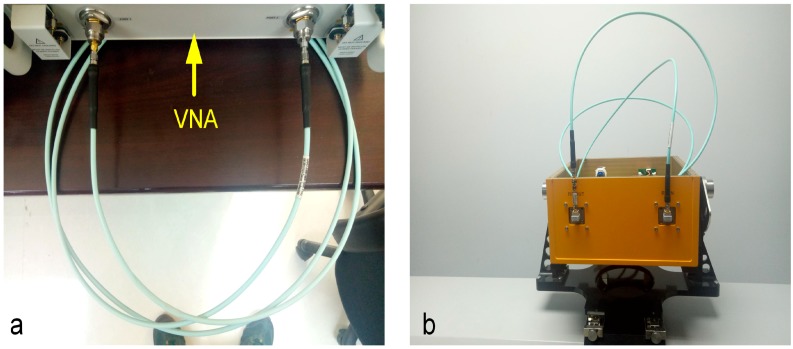
Phase stability test. (**a**) Testing cable delay and bending degree of cable with VNA; (**b**) The same cable will transmit interferometer directly to the receiving end for continuous sending and receiving multiple groups for phase stability test.

Several SAR images obtained at different times were irradiated at the same location using the zero-baseline repeated rail interference model to obtain deformation information. As is shown in Figure 6b, we assumed that the position of the CR1 target is *p*_1_ at time *t*_1_, and that it displaced at time *t*_2_ and changed to *p*_2_:

(6) $$\Delta R\cos\theta_{view}=\frac{R_{1}^{2}+\Delta R^{2}-R_{2}^{2}}{2R_{1}}$$

where *R*_1_ and *R*_2_ represent the antenna phase center distance to *p*_1_ and *p*_2_, respectively; *θ_view_* as shown in Figure 5b and 3D look in Figure 7 represents the azimuth angle of the target point relative to the GB-SAR.

By assuming dielectric characteristics of the pixel point n are the same for each observation time and by supposing the atmospheric contribution is removed. Displacement along the LOS can be retrieved according to Equation (7):

(7) $$\Delta d_{n}=R_{1}-R_{2}=-\frac{\lambda_{c}\cdot ∠(I_{1},I_{2}^{*})}{4\pi }$$

where ∠(·) denotes the phase information of the extracted complex image; *I*_1_, *I*_2_ represent complex matrixes collected before and after displacement, calculated from Equation (5); “*” is the complex conjugate operator. Equation (7) is a theoretical relation of LOS deformation obtained in the context of the spatial baseline of 0, and the few interference items considered. However, in non-continuous observation mode, the spatial baseline may not be 0. The complete more conventional interferometric phase expression is as follows:

(8) $$\Delta \varphi_{21}=\underset{f(B_{s})}{\underset{︸}{\varphi_{Geom}}}+\underset{f(B_{t})}{\underset{︸}{\varphi_{Defo}+\varphi_{Atmo}}}+\varphi_{Noise}-2k\pi$$

$\varphi_{Geom}$ refers to the terrain phase caused by the spatial baseline, and $\varphi_{Defo}$, $\varphi_{Atmo}$ refers to deformation and time phase change caused by the atmospheric influence; the continuous observation mode adopted by the system, close observation distance and spatial baseline of nearly 0 lead to that the image registration and terrain phase compensation become rarely necessary. $\varphi_{Noise}$ is noise and *k* is the integer ambiguity. Similar to InSAR, if the position of the vertical moving sensor during the two scanning periods can generate a spatial baseline $B_{s}$, so it does not exist in the experimental scene basically. But there is the temporal baseline $B_{t}$, and the time base-related component is still a part of the equation. In addition, there is also the observation noise phase caused by the instable system frequency and the change of target scattering characteristics. The phase change at the corner reflector serves as the major role studied in this experiment. Besides, the observation time is relatively short and the scattering characteristic of the object is relatively stable, so the noise phase is mainly caused by the instable system frequency, which mainly affects the phase relativity. And the measured transmitter-receiver signal noise ratio is 35 dB and thermal random noise decorrelation is negligible (|*γ*| > 0.999).

Before the outdoor experiment, the indoor phase stability experiment has been conducted. Different from [29], the phase stability of the interferometer with cables and converting elements with known time delay is tested in the experiment and the object is to get the inherent error of the main engine. The attenuation of the main engine through direct connection with cable is about 3 dB cable, and attenuator T-type network, 30 dB + 40 dB attenuator which may cause negligible phase variance. The calibrated vector network analyzer (VNA) is used to test the time delay of the cable, which is fixed in the load box of the main engine and its bending characteristic does not change during the whole operation process, and then the sum will be adopted. Then, one-dimensional imaging and one-dimensional interference were performed. Since the length of the whole process line and the bending degree of the line were unchanged, the phase change in the process was from the frequency change of interferometer itself. After repeated measurement of 100 sets of data to calculate the phase root mean square error, the interference deformation difference caused by system phase instability was determined to be ±0.034 mm.

The displacement generated by linear rail was kept within ±0.25*λ*. “Standardized coherence analysis” refers to the complex correlation between two complex image after registrations. The coherence can be derived from:

(9) $$\gamma =\frac{E\{I_{1}\cdot I_{2}^{*}\}}{\sqrt{E\{I_{1}\cdot I_{1}^{*}\}\cdot E\{I_{2}\cdot I_{2}^{*}\}}}$$

where represents the mathematical expectation. We used a similar IN-SAR image processing method for these calculations [30] and a movable search window. The coherence of each pixel is the calculation of Equation (9) for its adjacent *k* pixels. The search window is utilized to find the same targets in two images which are sufficiently consistent. Depend on the above processes, we obtained an amplitude image and primary displacement map as shown in Figure 6. Figure 6 shows data collected at 16:42 GMT+8. The rail sensor shows a true value of −2.0 mm. Figure 6a shows the relationship between “bright spots” and targets. The horizontal coordinate is the azimuth angle of each pixel, where CR1 has an azimuth span of −4.861° to −0.9722°. The pixel color represents the measurement value in the LOS direction. According to Equation (6), the true displacement is within the range of −1.826 mm to −1.8327 mm. Using LOS displacement instead of real displacement in this calculation affects the final results within 0.01 mm.

**Figure 8:**
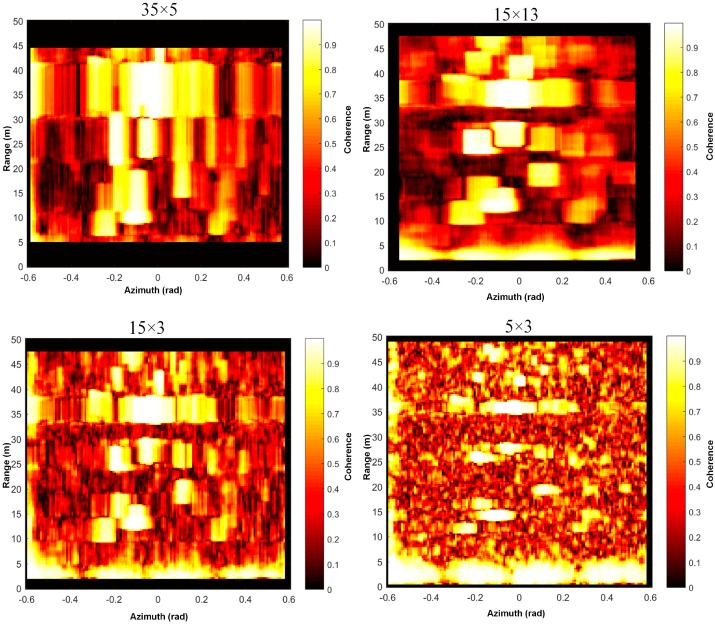
Different coherence window calculation size was tested. For example, “35 × 5” means in the range direction 35 pixels and in 5 pixels in the azimuth direction were used to calculate coherence via Equation (9).

When the rectangular window is used for calculation, it involves the selection of window length in azimuth direction and distance direction. The rectangular window of different sizes has been tried in this experiment, and the need for remote monitoring and rapid computing is taken into consideration for the initial equipment. The spatial resolution ratio 1km is taken as reference when calculating the coherence of window ratio. The two-dimensional coherence calculated is as shown in the figure below. It’s proposed in [15] that the coherence >0.6 could be used as a threshold, while it’s recommended using the coherence threshold of >0.9 in [19]. The appropriate coherent window can better reflect the phase quality parameters of the real scene, while the inappropriate one will cause great errors for the subsequent coherent scatterers analysis. In the same way, the two-dimension image also causes certain obstacles for judgment, which is mentioned in [29] that the way of image segmentation is firstly used to extract the permanent scatterers analysis from the real ground object, but later the PS pixels of object as well as the adjacent area, artefacts sometimes, are extracted. Actually, before starting the monitoring, the coherence two-dimension figure can be compared with the real terrain object, as the giant rock in Figure 3b, which should be shown as strong point of coherence in image, and its pixel range can also be observed through the spatial and image space analysis in Figure 4. Its real size should be roughly equivalent to the coherence pixels extensive scope. In fact, we cannot predict the parameters of the coherent window in advance. The monitoring area obtained by the coherent window has the largest available data, such as the parameters in the upper left corner of Figure 8, and with a certain coherence threshold. In this paper, the coherent threshold >0.8 is used to screen the deformation graph for displacement map at a certain moment.

**Figure 9:**
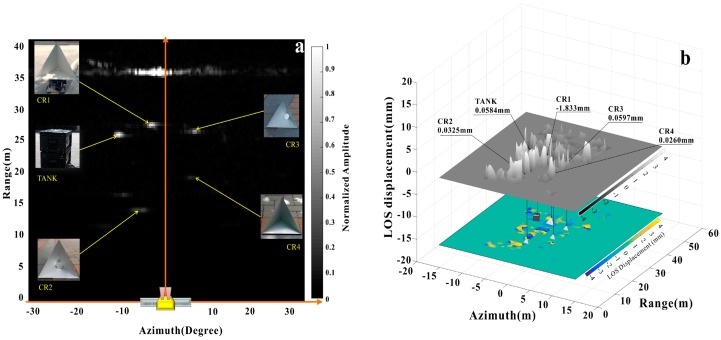
Typical SAR images (**a**) Amplitude image in polar coordinates; the “bright spots” are the corresponding targets; (**b**) The upper shows “surf plot”, displacements that were varied both in height and grayscale, the lower is the displacement map plotted as a color map.

The position of each target and the interferometric deformation measurement results are shown in Figure 9b. The presence of CRs enhanced the coherence of adjacent areas though some pixels of the image were displayed as outliers (deformation mutation or maximum/minimum values). 

Abnormal values may also have been caused by unordered tree oscillation, which can be effectively reduced in a long time series. It is difficult to recognize targets as-verified by 2D figures, so 3D matching methods merit further research.

## 4. Geometric Mapping and GB-SAR Displacement Optimization

### Geometric Mapping

**Figure 10:**
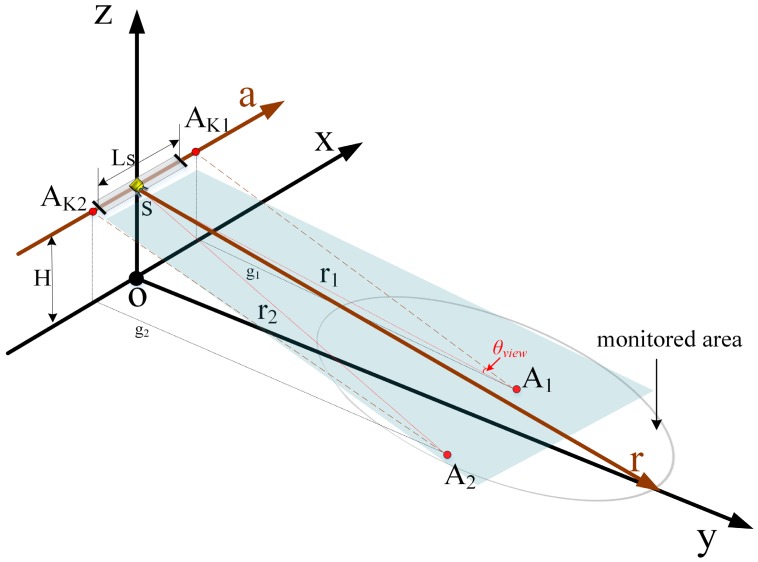
Geometric relationship between GB-SAR and 3D space. *A*_1_, *A*_2_ correspond to the CRs and *s* represents the phase center. The origin of the coordinate system was not selected as the location of TLS.

The zero-baseline working mode of GB-SAR does not directly include height information of the test site, which necessitates 3D conversion by geometric mapping with external DEM or DSM [27]. We used counterpart points to match the GB-SAR image with a TLS point cloud and projected deformation values onto it. Spatial rectangular coordinates were established as shown in Figure 10 and the rail position and feature points $A_{1}(x_{1},y_{1},z_{1}),A_{2}(x_{1},y_{1},z_{1})$ were drawn to represent CR2 and CR3 coordinates. *o* − *xy* is a plane parallel to the ground.

**Figure 11:**
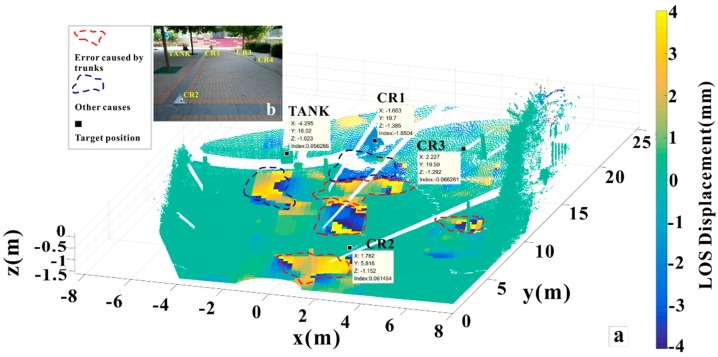
(**a**) Mapped 3D displacement map obtained at 16:42 GMT+8 colored by displacement value (in mm labeled as “index”) and the abnormal values were circled out; (**b**) Corresponding image of the scenario.

After calibration of the rail, the axis was approximately parallel to the ground. Point cloud data was obtained by TLS topographic mapping. GB-SAR system coordinates were determined as described in Section 2, with accuracy over 50 m of about ±1 mm [20]. s is the phase center of the GB-SAR array and s−ar is the cross section by range and azimuth direction. The plane rotates around the *s* − *a* axis as the target range changes. H is the rail axis distance to *o* − *xy*. We took the plane formed by the central point slant distance of the test site and the *s* − *a* axis as the reference plane for imaging. The obtained image is the projection image of each target in the reference plane. In the geometric mapping method, s corresponds to (0,0). In SAR image, $A_{1}$, $A_{2}$ respectively correspond to $A_{1}^{\prime }(r_{1},a_{1})$, $A_{2}^{\prime }(r_{2},a_{2})$; the problem to be solved is a mapping relation. As shown in Figure 10, $r_{1}$, $r_{2}$ are the slant ranges between $A_{1}$, $A_{2}$ and s. $A_{K1}$, $A_{K2}$ are the projection points on the *s* − *a* axis. The point cloud spatial resolution is higher than that of the GB-SAR image, with a multiple-to-one mapping relation. The range coordinates of SAR images are all positive, so the range direction matching relation is as follows:

(10) $$|A_{1}A_{K1}|\leq r_{1}\pm dr$$

(11) $$|A_{2}A_{K2}|\leq r_{2}\pm dr$$

where |·| represents the vector modulus, dr is as shown in Figure 4, i.e., the range direction sampling interval in the SAR image. $∠A_{K1}A_{1}s$, $∠A_{K2}A_{2}s$ respectively correspond to the azimuth angle in the SAR image. The azimuth matching relation expression is as follows:

(12) $$\vec{sA_{K1}}\leq a_{1}\pm da$$

(13) $$\vec{sA_{K2}}\leq a_{2}\pm da$$

where da is also shown in Figure 4, which represents the azimuth sampling interval of SAR images. We obtained the registration results shown in Figure 11 via the above matching rules. The trunk area shows deformation anomalies or discontinuities, so we conclude that random movement in the tree impacted the deformation measurement. There are other two deformation anomaly areas in Figure 11 marked by dark blue dashed circles. These areas should have been stable during the monitoring process (displacements < 1 mm) but showed changes, likely by mutations in coherence and phase discontinuity due to the addition of artificial objects. In the natural state, the phase change of ground objects should be continuous. 

The abnormal areas in Figure 11 can be verified through TLS displacement measurement. Currently effective method utilizes surface fitting of point sets and calculation of the gap between parallel planes, which is well performed in composite structures deformation extraction [31]. Main objective is to optimize the projection error from the point set to the fitting plane There are several methods like least-squares plane fitting, principal component analysis (PCA) normal vector estimation, singular value decomposition (SVD) [32]. SVD merits better numerical stability than the former two methods, with higher accuracy. For the detailed calculation process of SVD, can be referred to [33], and is also worked as “svd” function in MATLAB. Following are main steps for surface reconstruction:

- The fitted plane M should go through the mean of the points set $xyz_{mean}$.
- The point set of CRs or floor tiles within abnormal areas is selected, denoted as Data.
- Subtract the point cloud data with the average point to form a centered plane.
- The centered plane is subjected to SVD to get *U*, Σ and *V^T^*. U_m__*m_ and *V*_3*3_ are unitary matrices, “*T*” represents transposition. Σ is a positive semi-definite diagonal matrix, the singular values are elements on the diagonal. MATLAB code is [*U*,Sigma,*V*] = *svd*(*centeredplane*).
- The smallest singular value corresponds to the direction of the most concentrated distribution of the point set, which is the normal vector direction of the plane.

**Figure 12:**
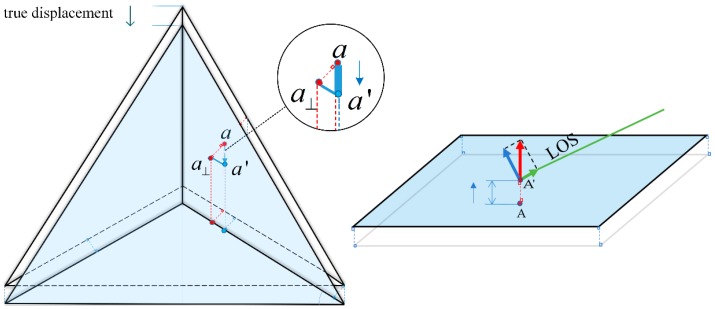
TLS CR1 and tiles displacement calculation. For CR1, the blue colored plane is the plane after moved. *a* and *a*′ are on different planes, which respectively represents the gravity center of the two planes before and after moved. Right part shows tiles displacement, GB-SAR measurement is the true displacement vector (the red arrow) projected onto the LOS direction (the green arrow).

Then fit according to the plane equation:

(14) M=−(d+a∗x+b∗y)/c

where *a*, *b*, *c*, *d* are all undetermined coefficients a=V(1,3); b=V(2,3); c=V(3,3); $d=-dot([a,b,c],xyz_{mean})$, “dot” means dot product, [·] denotes for a vector and inside components. Then go on with a geometrical relationship analysis, as shown in Figure 12, the above right side of CR1 and ground tiles was fitted by the above method and two groups of data before and after CR1 movement were obtained by TLS scanning. The gravity center or isosceles right triangle of the two CR1 planes is |a| and $a^{\prime }$, and the $\vec{aa^{\prime }}$ vector is the real deformation to be solved. Let $a_{⊥}$ be the vertical projection of a onto the moved plane (blue colored), ${|aa}_{⊥}|$ is the distance between two parallel planes, and $∠aa^{\prime }a_{⊥}$ is the angle between the true displacement direction and two parallel planes. When CR1 was placed parallel to the ground, plane $∠aa^{\prime }a_{⊥}$ was parallel to the bottom, $∠aa^{\prime }a_{⊥}$ was 45°, and $\left|aa^{\prime }|=\sqrt{2}|aa_{⊥}\right|$. Similarly, for the floor tiles, which are closely arranged, the tiles can be considered only deformed in the vertical direction. A and $A^{\prime }$ are the gravity center, and the LOS displacement for GB-SAR was $\theta_{\mathrm{LOS}}\cdot \left|{AA}^{\prime }\right|$, $\theta_{\mathrm{LOS}}$ represents the LOS incident angle for A and $A^{\prime }$, which can be calculated from the point cloud according to Figure 10.

**Figure 13:**
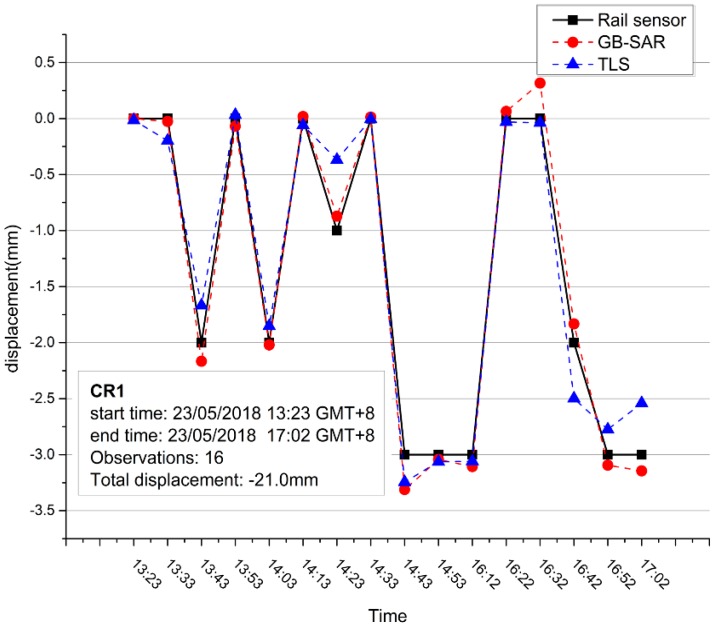
Comparison between rail sensor value, GB-SAR LOS interferometry, and TLS measurement.

Table 3 shows the GB-SAR and TLS comparison at P1 and P2 calculated upon method mentioned above, which we P1 and P2 were shown in Figure 11a. Undoubtedly, they should with very small cumulative displacement. However, as shown in Figure 13, the GB-SAR results accord well with the rail sensor values. And GB-SAR accuracy is slightly higher than the TLS measurement at CR1:GB-SAR average error is 0.01687 mm and TLS average error is 0.03881 mm. In this case, if some areas are stable and possess coherence as high as the CRs in lengthy time series, they will produce high signal-to-noise (SNR) echo and become “coherent scatterers”, surely merit credible measured value. 

“Coherent scatterers” selection is introduced based on the temporal coherence and DA analysis in order to eliminate the abnormal regions in displacement map. The criteria for coherent scatterers are based on the amplitude of the pixels in the coherence map. The key parameter we utilized is dispersion of coherence amplitude (DA), in case of error accumulation due to permanent scatterer screening. Low DA value indicates a “good” pixel, that is, a higher SNR. For *Q* numbers of coherence maps, DA can be calculated by following equations:

(15) $$DA(i,j)=\frac{Stdev[A_{1}(i,j),\cdots ,A_{Q}(i,j)]}{MA(i,j)}$$

(16) $$MA(i,j)=\frac{1}{Q}\sum_{k=1}^{Q}A_{k}(i,j)$$

where *Stdev*[·] represents standard deviation. *A_k_*(*i*,*j*) denotes for the amplitude of pixel at (*i*,*j*) in *k*th coherence map. *MA*(*i*,*j*) stands for mean amplitude. The threshold of the DA value needs to be determined, that is, it needs to weight between phase quality and the density of available deformation measurements. 

**Figure 14:**
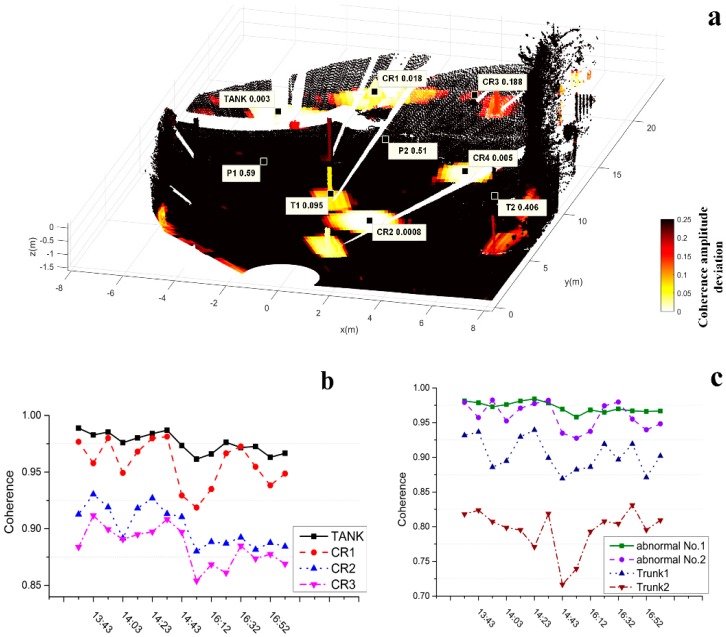
3D coherence analysis (**a**) DA map projected to point cloud, colors identify the DA less than 0.25. DA values of some points corresponding to points in (**b**,**c**) are shown. (**b**) Coherence curve of artificial targets over time; (**c**) Anomalous areas in Figure 7 where coherence changes with time, abnormal No. 1 and abnormal No. 2 correspond to P1 and P2 in panel (**a**).

**Figure 15:**
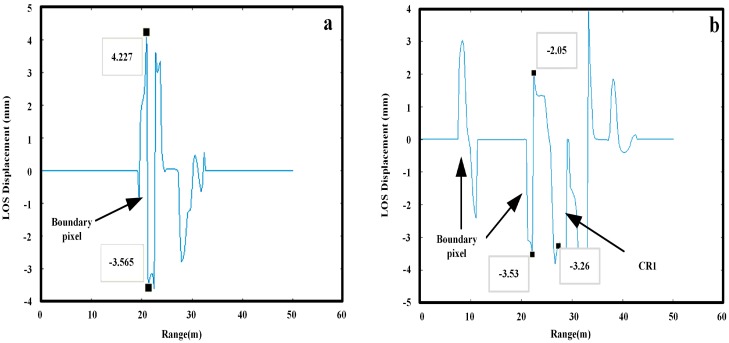
1D profiles including abnormal discontinuous boundary and CR1. The abnormal bumps are in the range direction of the displacement map (**a**) An example profile contains boundary pixel; (**b**) Profile contains boundary pixel and CR1.

We projected a DA analysis map onto the DSM as shown in Figure 14a. A total of 15 coherence maps were collected successively, and the “highlighted” part indicates DA less than 0.25. Surely, the abnormal areas represented by P1 and P2 can be filtered. Then, we conducted coherence analysis and set a threshold value based on a pixel coherence value higher than 0.9 in a single image. Figure 14b,c show temporal coherence changes of the artificial targets and natural targets, respectively. The CR2 curve shows strong coherence overall, but there are several moments at which coherence was below 0.9. A poor electromagnetic wave incidence angle may cause the corresponding pixel coherence lower than 0.9. The two curves indicate that targets with the same physical properties may vary in coherence by different imaging geometry. Points T1 and T2 in the Figure 14a correspond to Trunk1 and Trunk2 in Figure 14c, reflecting the coherence vibration of the trunks. However, this threshold is not effective as-applied to the other two abnormal deformation areas. P1 and P2 positions also have strong coherence over a long period of time due to the addition of artificial targets. Locations with high deformation reliability (i.e., coherent scatterers) were effectively screened via the above process. In the next, an appropriate filtering algorithm was sought to manage the outliers due to DA and temporal coherence dual-threshold screening. But the screening method may cause discontinuity boundary as shown in Figure 15. The 1D sections containing abnormal boundary points. The horizontal coordinate represents a range value and the vertical coordinate a deformation value. Abnormal points present as severe “jump-changes” with positive and negative peaks. The deformation of CR1 is marked in Figure 14b. Linear displacement has no corresponding peak in the opposite direction, so a linear window filtering algorithm can be used to optimize the results.

**Figure 16:**
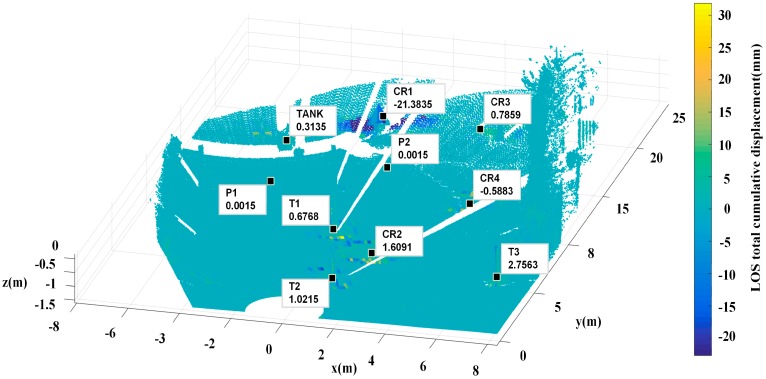
Filtered 3D total cumulative displacement map. Abnormal areas shown in Figure 8 were filtered (P1 and P2). T1, T2 and T3, which within highlighted regions in Figure 11a, stand for the trunks.

Linear filtering is utilized to reduce boundary discontinuity or boundary anomaly pixels to prevent error accumulation in longer time series. Uniform sized windows of 3 × 3, 5 × 5, 7 × 7, and 9 × 9 were separately tested. Oversized window may affect the observation value at CR1, but undersized window is insufficient to filter abnormal values. The total cumulative deformation measurements of 15 data sets at each point (mm) are shown in Figure 16, the artificial targets and the trunks work as natural targets total cumulative displacements were labeled out. There were still displacements around P1 and P2 points that may be attributable to pedestrians crossing through the experimental setup, but they deformed slightly. 

**Figure 17:**
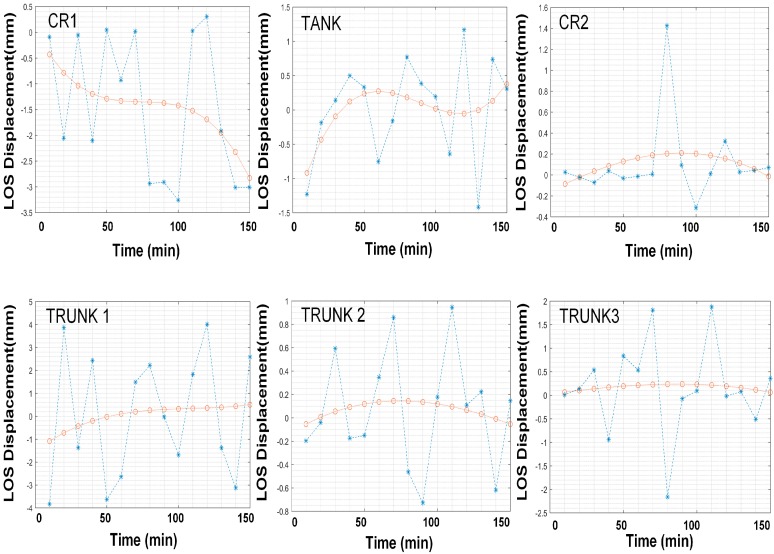
Displacements comparison of selected points in time series, T1, T2, and T3 in Figure 16 correspond to TRUNK1, TRUNK2, and TRUNK3 respectively.

Figure 17 shows the dashed line (blue) of GB-SAR measurements at points CR1, CR2, TANK, T1, T2, and T3 as well as the third-order polynomial fitting curve (orange colored). The CR2 and TANK variables were consistent with our predictions. The CR2 data moved slightly in a certain track which is indicated as a sharp peak in the graph. Time series displacements at T1, T2 and T3 was positive and negative, respectively.

## 5. Discussion

**Figure 18:**
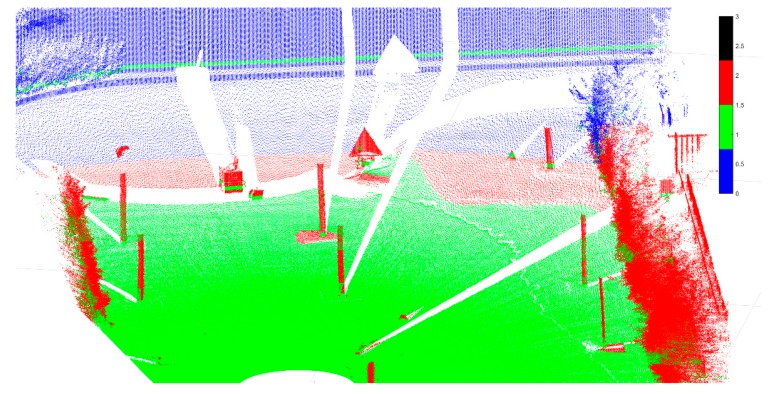
Preliminary results of automatic classification of DSM point cloud, the target is to select the coherent points target in the red region, green represents the ground, and blue represents the unclassified object.

Compared with the method that the PS point was selected based on the two-dimensional image [29], while coherent scatters are selected in this paper to select the PS the point through point cloud classification. The accurate classification of the point cloud is the process of identifying the attributes of the point cloud which is of great significance in improving the reliability and automation of the modeling process. This paper also classifies the point cloud as shown in Figure 18. RANSAC algorithm is mainly used in this process [34], which is divided into three processes: point cloud acquisition, point cloud segmentation and point cloud classification. The point cloud segmentation mainly divides the main monitoring targets and selects coherent scatters from these target point clouds (red areas). Through the accurate segmentation of the point clouds, CR1 and TANK can be separated from the nearby ground, which will not be described in detail here. Only the initial automatic classification result of the point cloud is displayed here. The red color is the area where the coherent scatter point is to be selected, the green area is the ground, and the blue and black correspond to the unclassified result.

We can see from Figure 16 the discontinuous boundary and the result without phase correction. There is a strong coherence between the discontinuous boundary, the area around the artificial targets and Da, but the side lobes of azimuth are strong, long and highly coherent due to the influence of artifacts and now they are not the target of observation but the pseudo-target, however, pseudo-targets are not negligible details.

The current effective early warning analysis of landslides and collapses is mainly based on the deformation threshold and the method of adjacently exceeding the threshold pixel points [35]. The pseudo-targets make the dangerous deformation area expand, which will cause the error analysis of the slope instability and false alarms. The solution is to select the peak position within a certain area near the peak of the point target. That is to say, the peak position is accurately demarcated from the two-dimensional deformation map. The method can be achieved through the back analysis of point cloud matching. Thus, the method of accurate 3D mapping is very important for us. In this paper, the control point method is used to confirm the accuracy of the matching result. 

According to the DA analysis of Figure 14, the threshold value can be further increased and DA < 0.25 is a recommended result [29]. But for this paper in determining whether the low DA value region is accurately matched to the three-dimensional point cloud, this threshold value is still too high. We believe that lowest DA value of a two-dimensional image should focus on the true scattering center of each artificial target. The displacement of each artificial targets does not exceed a range resolution cell (Δ*d_cumulate_* < *δ_r_*, Δ*d_cumulate_* representing the cumulative displacements). In the analysis process, this paper proposes to use the method of “large grid”. The data block in the new grid is equivalent to the “large pixel”. The construction of the large pixel is in line with the law of slope monitoring and block analysis because the previous analysis mode is based on the pixels of the two-dimensional image, such as [15,36], which is not obvious enough for real-world spatial deformation analysis. The three-dimensional projection scheme of point cloud proposed in [18,19] is very effective for the analysis of large-scale and large-area Slope instability. But sometimes in order to accurately locate small-scale collapses and to find the creep characteristics of the slope in advance, we need the accurate grid analysis. 

**Figure 19:**
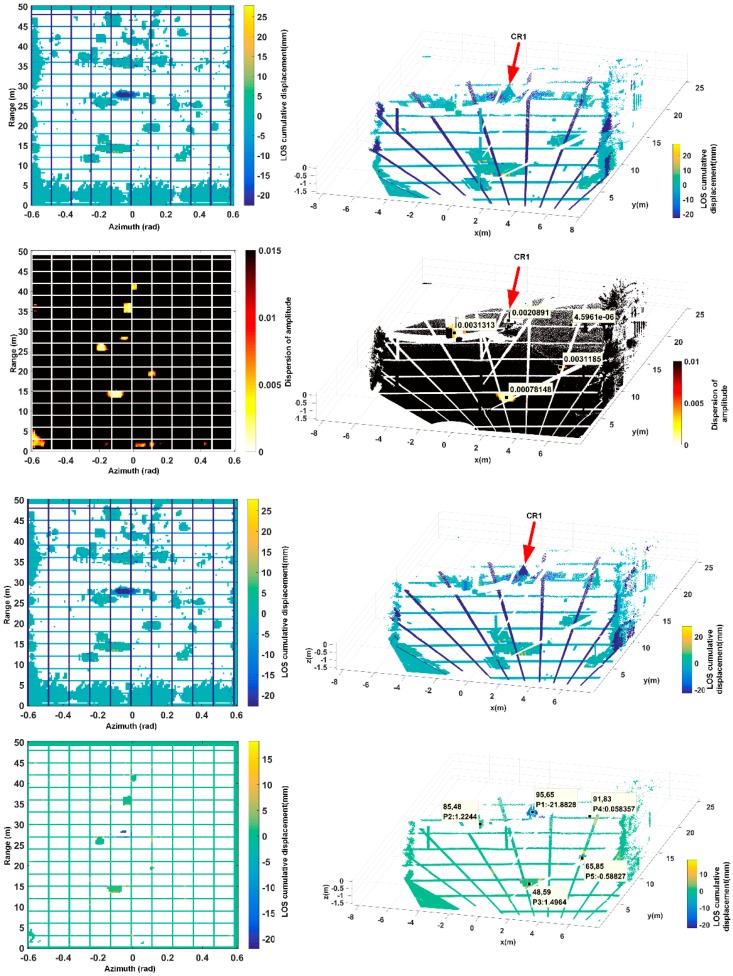
In the first line, two figures show the 2D displacement map and 3D mapping grid relationship, but the 3D mapping result is not corrected. And in the second line, the two figures show the DA 3D mapping, and the pixels whose DA are less than the threshold of DA < 0.01 are shown. In the third line, two figures show the corrected 3D mapping result. The last line shows only the pixels that DA < 0.01 are shown, which is used to select ground control points for atmosphere correction.

As shown in Figure 19, the experimental scene based on the above requirements is divided into partial “large pixels”, which are divided according to the azimuth spatial extension length and the range direction extension length of each target in the scene (considering the artifact length) to make one target occupied by one or two Large pixels. In order to distinguish the target pixel in a more intuitive way, continuing to change the color of the grid boundary. The farther away from the radar, the deeper color of the target “big pixel”. In the DA analysis method shown in the two figures of the second row, the threshold value of DA is reduced to 0.01, at which, the two-dimensional pixel of low DA value corresponding to CR1 has been reduced to several ones. The translation and scaling are performed to adjust the distance and the azimuth distance, which can achieve accurate matching. The adjustment method is simple and thus will not be described in detail here. The result of the exact match is shown in the right figure of the second row of Figure 19. From the DA value shown by the cursor in the figure, the DA value of the artificial target is very small. The two pictures of the third row show the matching result corrected by 3D mapping. but the matching result is not rescreened by DA threshold value this time. It shows the result that each frame of the image is filtered by the threshold value of correlation > 0.8. the two pictures of the fourth row show the final result, at which the artifact extension of CR1 is effectively suppressed and the problem of discontinuous boundary pixel has been also be effectively reduced.

**Figure 20:**
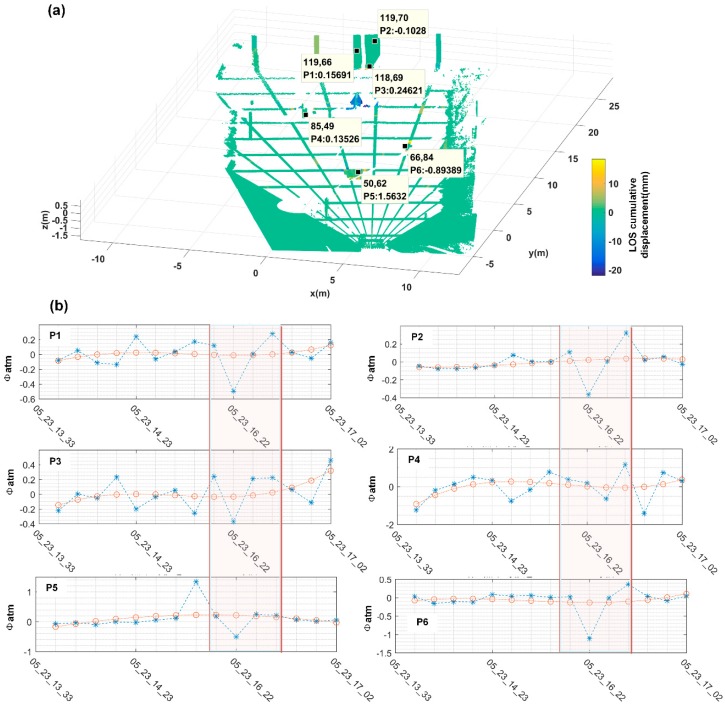
(**a**) For the GCP points selected based on the DA < 0.1 and only on the artificial target, there are 6 in total, among which the displacement of P5 points is artificially shifted at a certain moment. (**b**) In the red color frame, it was found that each GCP point had a strong correlation, and the atmospheric disturbance occurred during this period was comprehensively determined. The farther away from GB-SAR, the more severe the target disturbance, indicating that the atmospheric disturbance in the small region was more consistent, and it could be considered that it changed linearly with distance.

**Figure 21:**
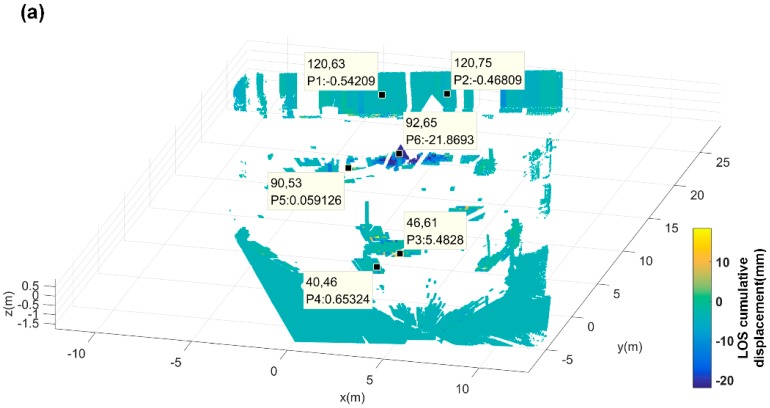

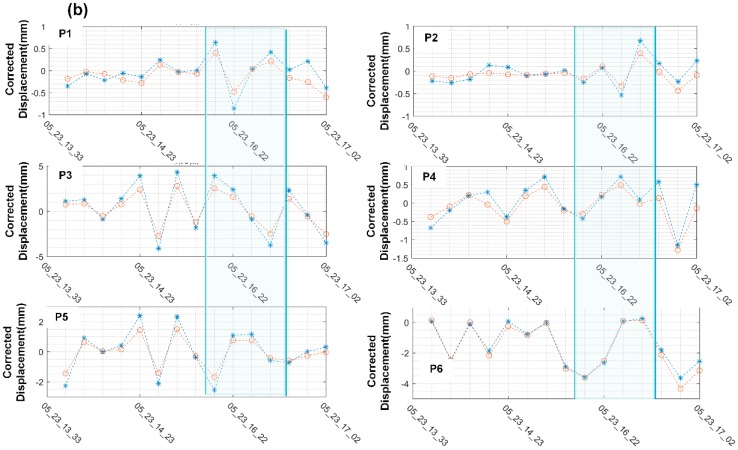
(**a**) Additional selected relative unstable points other than stable points, according to DA < 0.25 and evaded artifact effect; (**b**) The data of each point in the blue box showed some improvement, closer to zero. However, due to the system frequency instability, it was +/− 0.34 mm, atmospheric correction had limited effect in this experiment.

We choose several coherent scatterers in three-dimensional space. The reason why we don’t call them permanent scatterers is that we do not have more than 30 frames of images for analysis according to the proposal of [29,36]. However, it is still of great significance to use more coherent points to remove atmospheric impacts in a short time. For example, in the condition of landslide emergency monitoring, if the appropriate monitoring position is selected for correction, monitoring parameters and conditions need to be determined rapidly and adjusted timely. At this point, the method in this paper can be used for analysis. Specifically, C numbers of stable pixels (Δd=0) can be selected in the low DA region through the above 3D mapping method and coherent scatterers analysis. After the system noise was corrected by the method shown in Figure 7, the phase change caused by the change of back scattering characteristics was ignored. At time *p*, let the atmospheric phase as $\varphi_{pAtm}(i_{c},j_{c})=k_{pc}r(i_{c},j_{c})$ and the phase changes denoted by $\Delta \varphi_{pq}(i_{c,}j_{c})$ of each stable points from time *p* to time *q* are all considered as atmospheric delay:

(17) $$\Delta \varphi_{pq}(i_{c,}j_{c})=k_{qc}r(i_{c},j_{c})-k_{pc}r(i_{c},j_{c})$$

(18) $$k_{pqc}=k_{qc}-k_{pc}=\frac{\Delta \varphi_{pq}(i_{c},j_{c})}{r(i_{c},j_{c})}$$

(19) $$d_{k}=\max(k_{pqc})-\min(k_{pqc})$$

where $k_{pqc}$ is atmosphere delay factor, and it reflects the change of atmospheric delay per unit length of ground control points from time *p* to time *q*. $d_{k}$ stands for the degree of regional atmospheric disturbance. When $d_{k}$ is small, it indicates that the atmospheric delay variation of multiple control points is consistent. And when $d_{k}$ is large, it indicates that the atmospheric delay change in the monitoring area from p to q is not uniform. Then set a threshold $d_{kL}$, when $d_{k}>d_{kL}$, the image would be eliminated. For three-dimensional point cloud, since the spatial resolution is higher than GB-SAR, Equation (18) calculation is relatively more accurate. The atmospheric disturbance factor of the relative unstable pixel (i,j) is calculated according to the inverse distance weighting (IDW) of all the stable pixels, and the weight is calculated as follows:

(20) $${\bar{K}}_{pq}(i,j)=\sum_{c=1}^{C}\lambda_{i}K_{pqc}$$

(21) $$\lambda_{i}=(1/d_{i})/(\sum_{i=1}^{C}1/d_{i})$$

where C means the stable points which meet $d_{k}<d_{kL}$, $d_{i}$ is not calculated by Equation (19), it stands for the distance between unstable points to the *i*-th stable point. And $\lambda_{i}$ represents the *i*-th stable point weight at the unstable point. ${\bar{K}}_{pq}(i,j)$ means the atmosphere delay factor at the unstable point (i,j). By (17)–(21), the atmospheric delay is calculated. Ground control points (GCPs) are first selected through 3D mapping correction and DA < 0.01. As shown in Figure 20a, the low DA values were screened out at pixels around CR2 and CR4 (P5 and P6) and TANK (P4), and with other three points on the stable parapet wall in the illuminated area (P1, P2 and P3). The temporal of interference phase curve during monitoring time of each selected GCPs is shown in Figure 20b. Then, the relative unstable points are selected, and the unstable points are from the points selected by DA < 0.25, as shown in Figure 21a. The comparison between the two points before and after atmospheric correction of each unstable point is shown in Figure 21b.

## 6. Conclusions

In this study, we integrated TLS, CR, and GB-SAR interferometry sensors to validate deformation in a controlled field. A novel quantitive GB-SAR sensor deformation monitoring experiment was established comprised of artificial targets layout strategy, displacement extraction of GB-SAR and comparison with TLS surface reconstruction methods. And more, the coherent scatterers selection and filter method for discontinuous boundary were detailed which may help to ultimately achieve sub-millimetric accuracy over the course of continuous monitoring and more reliable results. GB-SAR has all-weather monitoring ability and accuracy which is unmatched by other methods; TLS is preferred for its rapid and high precision surface reconstruction. Combining these two technologies with CRs allows for highly effective data fusion for more comprehensive deformation monitoring. 

This study was conducted in close range (<50 m). An experimental scheme using far-field monitoring (>500 m) merits further study, as TLS laser beams are likely more divergent over greater distances. If the monitoring distance increases by 100 m, the beams we used in this study would diverge more than 27 mm and a reflective sticker would no longer recognize CR coordinates. However, a smoother surface material and higher reflectivity material (or coating) could be adopted in the CR design to account for this. CRs may also be reshaped for higher volume to be consistent with the GB-SAR resolution cell size.

TLS systems can reconstruct the resolution cell of surface models to efficiently and effectively identify deformation. The deformation generating rail used here could also be improved by using dc batteries or a solar power supply, as is widely used in buried earthquake monitoring instruments for long-term monitoring. The rail can simulate linear deformation over a longer time interval when it is sufficiently powered. Furthermore, although natural targets generally exhibit nonlinear movement and have properties divergent from the CR, this study using artificial controlled deformation targets still has research significance.

In addition, in our experimental scheme, a phase unwrapping problem was created when the displacement of a certain point exceeded the maximum recognizable limit of a single track (±0.25*λ*). Phase wrapping methods are mainly realized by the transplantation of IN-SAR data processing algorithms, which require strong phase continuity. Artificial targets can cause phase discontinuity problems, but there is currently no algorithm that is insensitive to phase continuity.

Our results indicate that the fusion of GB-SAR with other instruments is a very effective approach to GB-SAR interferometry. We hope that the results presented here may lead to an effective scheme for the practical engineering of early warning models based on various modes of deformation.

## Figures and Tables

**Table 1:** Linear System Parameters.

Parameter	Preference	Description
Positional accuracy	0.1 mm	Positional deviation determined by sensor
Precision	0.1 mm	Position deviation under constant conditions
Displacement accuracy	≥1 mm	Minimum rail displacement
Sliding table load	≤25 kg	

**Table 2:** GB-SAR Interferometer Characteristics.

Characters	Value
Radar center frequency *f_c_*	17.25 GHz
Radar bandwidth *B*	500 MHz
Synthetic aperture length *L_s_*	1 m
Linear scansion point number *N*	126
Antenna gain	0 dB
Transmitted power	33 dBm
Polarization	VV
Target distance	0–40 m
Measuring time per image	10 min
Number of transmitted frequencies *K*	10,001

**Table 3:** GB-SAR and TLS displacement measurement at *p*_1_ and *p*_2_.

Time	GB-SAR Displacement at *p*_1_/mm	TLS Displacement at *p*_1_/mm	GB-SAR Displacement at *p*_2_/mm	TLS Displacement at *p*_2_/mm
16:52	−3.0289 mm	0.392 mm	2.479	0.239
17:02	2.039 mm	0.013 mm	2.405	0.336
